# sFlt-1 and CA 15.3 are indicators of endothelial damage and pulmonary fibrosis in SARS-CoV-2 infection

**DOI:** 10.1038/s41598-021-99470-y

**Published:** 2021-10-07

**Authors:** Marilena Greco, Salvatore Suppressa, Roberta Assunta Lazzari, Fernando Sicuro, Carmelo Catanese, Giambattista Lobreglio

**Affiliations:** 1 Clinical Pathology and Microbiology, Vito Fazzi General Hospital ASL-Lecce, 73100 Piazza Muratore, Lecce, Italy; 2 Intensive Care Unit, Vito Fazzi General Hospital ASL-Lecce, Lecce, Italy

**Keywords:** Biomarkers, Diseases, Health care, Medical research

## Abstract

COVID-19 pandemic led to a worldwide increase of hospitalizations for interstitial pneumonia with thrombosis complications, endothelial injury and multiorgan disease. Common CT findings include lung bilateral infiltrates, bilateral ground-glass opacities and/or consolidation whilst no current laboratory parameter consents rapidly evaluation of COVID-19 risk and disease severity. In the present work we investigated the association of sFLT-1 and CA 15.3 with endothelial damage and pulmonary fibrosis. Serum sFlt-1 has been associated with endothelial injury and sepsis severity, CA 15.3 seems an alternative marker for KL-6 for fibrotic lung diseases and pulmonary interstitial damage. We analysed 262 SARS-CoV-2 patients with differing levels of clinical severity; we found an association of serum sFlt-1 (ROC AUC 0.902, decision threshold > 90.3 pg/mL, *p* < 0.001 Sens. 83.9% and Spec. 86.7%) with presence, extent and severity of the disease. Moreover, CA 15.3 appeared significantly increased in COVID-19 severe lung fibrosis (ICU vs NON-ICU patients 42.6 ± 3.3 vs 25.7 ± 1.5 U/mL, *p* < 0.0001) and was associated with lung damage severity grade (ROC AUC 0.958, decision threshold > 24.8 U/mL, *p* < 0.0001, Sens. 88.4% and Spec. 91.8%). In conclusion, serum levels of sFlt-1 and CA 15.3 appeared useful tools for categorizing COVID-19 clinical stage and may represent a valid aid for clinicians to better personalise treatment.

## Introduction

The novel severe acute respiratory syndrome coronavirus 2 (SARS-CoV-2), responsible for the coronavirus disease 2019 (COVID-19) pandemic, has led to a rapid and extensive worldwide increase in hospitalisations for interstitial pneumonia, with several complications such as thrombosis, endothelial injury and multiorgan disease.

SARS-CoV-2 infection targets nasal, bronchial, epithelial cells and pneumocytes which express the angiotensin-converting enzyme (ACE2) receptor^1^ for the viral spike (S) protein. Inflammatory signalling molecules are released by infected cells and alveolar macrophages; T lymphocytes, monocytes and neutrophils are recruited on the site of infection and cytokines release enhances inflammatory response. Diffuse alveolar interstitial thickening and interstitial mononuclear inflammatory infiltrates and edema develop, and typical ground-glass opacities appear on CT imaging. Autoptic analysis of lung tissue showed marked fibrotic lung parenchyma remodelling, fibroblast proliferation and airspace obliteration^2^.

Endotheliitis and presence of viral elements inside endothelial cells has been shown in postmortem analysis of different organs of Covid-19 patients^3^. SARS-CoV-2 infects capillary endothelial cells due to the expression of ACE2 receptor also by endothelial cells^4^. Covid-19 endotheliitis has been suggested as an explanation of the systemic impaired microcirculatory function with consequent pro-coagulant state^5^ and clinical sequelae in SARS-CoV-2 infected patients^3^.

Common laboratory abnormalities of individuals with COVID-19 include lymphopenia, elevated inflammatory markers (e.g., C-reactive protein, ferritin, tumor necrosis factor-α, IL-1, IL-6), and abnormal coagulation parameters (e.g., elevated D-dimer, prolonged prothrombin time, thrombocytopenia, low fibrinogen)^6^. Increase of Red Cell Distribution Width (RDW) has been associated with disease severity in hospitalised adults with SARS-CoV-2 infection, previously published RDW thresholds of ≥ 14.5% (RDW-CV) or ≥ 47 fL (RDW-SD) have been associated to increased risk of death^7–9^.

However, no specific laboratory marker has been so far established for COVID-19 and for evaluation of the risk of COVID-19 pulmonary fibrosis and endothelial dysfunction.

In the present study we analysed blood samples from 262 patients who had tested positive for SARS-CoV-2 infection at different time from diagnosis and with differing clinical severity status and we utilised sFlt-1 and CA 15.3 as circulating markers of endothelial damage and pulmonary fibrosis, respectively. Serum sFlt-1 being the soluble form of Vascular Endothelial Growth Factor receptor (sVEGFR) which binds and antagonizes Vascular Endothelial Growth Factor (VEGF) and Placental Growth Factor (PlGF) signal^10^ and it has been demonstrated to promote endothelial dysfunction, notably during preeclampsia^11^. According to our previous results, sFlt-1 is as a biomarker of endothelial injury associated to bacterial sepsis and sepsis severity^12^ and previous evidence showed it increased in COVID-19^13,14^. The CA 15-3 is as an alternative marker for KL-6 (serum Krebs von den Lungen-6) in fibrotic lung diseases which increases in pulmonary interstitial damage, fibroblast activity and progression to fibrosis of the lung^15,16^ and might be of great interest when studying the effects of COVID-19.

## Materials and methods

### Patients

The present study has been conducted on 262 patients hospitalised in the COVID Division of the Vito Fazzi Hospital of Lecce (Italy), 227 survived and 35 died during the hospitalisation; 101 not hospitalised healthy individuals were used as controls (Table 1).

**Table 1:** Characteristics of SARS-CoV-2 patients and controls.

	COVID-19 patients			Control patients
	All	Survivors	Dead	
N Age (years)	262 66.6 $\pm$ 2.1	227 68.9 $\pm$ 1.4	35 72.1 $\pm$ 4.4	101 60 $\pm$ 1.1
Males Age (years)	155 62 $\pm$ 1.1	130 62.8 $\pm$ 1.9	27 68.9 $\pm$ 4.9	47 59.1 $\pm$ 1.4
Females Age (years)	107 72.1 $\pm$ 1.4	97 74.5 $\pm$ 2.0	8 80.7 $\pm$ 9.3	54 60.0 $\pm$ 1.6
Disease severity in hospitalized patients:				
Dyspnea		41 (18%)	25 (71.4%)	
Respiratory Rate (> 30 breaths/min)		34 (14.9%)	19 (54%)	
Blood Oxygen Saturation (< 93%)		47 (20.7%)	23 (65.7%)	
PaO_2_:FiO_2_ (< 300 mm Hg)		53 (23.3%)	29 (82.8%)	
Lung Field Infiltrate (> 50%)		100 (44.1%)	26 (72.3%)	

Serum samples were collected in the Clinical Pathology and Microbiology Laboratory of the Vito Fazzi Hospital of Lecce (Italy); selected patients included individuals positive for SARS-CoV-2 infection (without bacterial co-infection) and healthy individuals undergoing routine clinical investigation as control patients. All leftover serum specimens were anonymised and stored at − 80 °C until further use. Sampling of COVID-19 patients was performed over a time frame of 0–28 days during hospitalisation, from first oro-nasopharyngeal swab positive for SARS-CoV-2 RNA.

The diagnostic criteria for COVID-19 followed the interim or 7th edition guideline of The National Health Commission^17^ which mainly included epidemiological history, clinical symptom, thoracic CT examination, confirmed presence of SARS-CoV-2 RNA by real-time reverse transcription polymerase chain reaction (RT-PCR) of oro-nasopharyngeal swab.

The severity of the illness was based on the presence of dyspnea, respiratory rate of 30 or more breaths per minutes, blood oxygen saturation of 93% or less, a ratio of PaO_2_:FiO_2_ of less than 300 mm Hg, an infiltrate in > 50% of the lung fields. Indicator of severe disease were marked tachypnea, hypoxemia, abnormal lung findings (Table 1) and abnormal laboratory results listed in Table 2^18,19^.

**Table 2:** Hematochemical parameters in survived and deceased COVID-19 patients in relation to sFLT-1 quartiles (I–IV).

		I	II	III	IV
		91–99.7	99.8–117	117.1–142	> 142.1
**Lymphocytes (10**^**3**^**/µL)**	*Survivors*	1.6 ± 0.1	1.2 ± 0.1	1.2 ± 0.1	1.1 ± 0.1
	*Dead*	–	0.3 ± 0.1	0.5 ± 0.1	0.9 ± 0.1
**Neutrophils (10**^**3**^**/µL)**	*Survivors*	4.8 ± 0.4	5.8 ± 0.5	7.5 ± 0.9	9.4 ± 1.2
	*Dead*	–	12.9 ± 3.5	9.7 ± 3.9	9.5 ± 0.9
**RDW-SD (fL)**	*Survivors*	45.3 ± 1.1	44.6 ± 1.2	45.8 ± 0.9	45.3 ± 1.1
	*Dead*	–	50.0 ± 2.2	45.5 ± 2.3	45.4 ± 1.7
**D-Dimer (ng/mL FEU)**	*Survivors*	3997.6 ± 1142.9	1389.6 ± 183.4	7306.2 ± 2575.0	1863.6 ± 397.8
	*Dead*	–	1783.0 ± 753.1	34,956.0 ± 16,886.8	2028.7 ± 465.1
**CRP (mg/L)**	*Survivors*	34.5 ± 7.2	51.9 ± 9.7	84.4 ± 13.8	118.9 ± 19.1
	*Dead*	–	117.7 ± 61.2	193.6 ± 50.4	110.9 35.9
**LDH (U/L)**	*Survivors*	198.2 ± 15.0	234.2 ± 25.5	265.2 ± 20.2	313.8 ± 31.9
	*Dead*	–	276.0 ± 75.2	341.0 ± 39.7	401.2 ± 40.5
**Ferritin (ng/mL)**	*Survivors*	569.7 ± 59.5	793.2 ± 79.0	1012.4 ± 126.8	1362.9 ± 216.8
	*Dead*	–	1697.0 ± 1224.7	1927.8 ± 738.8	1643.1 ± 606.0
**PCT (ng/mL)**	*Survivors*	0.1 ± 0.05	1.5 ± 1.1	0.7 ± 0.3	1.6 ± 0.7
	*Dead*	–	1.5 ± 0.5	0.5 ± 0.2	1.9 ± 0.8

### Real-time PCR assay for SARS-CoV-2 RNA detection

Oro-nasopharyngeal swabs in UTM were fully automated processed for extraction, amplification and result analysis with ELITe InGenius (ELITechGroup, Torino, Italy) according to manufacturer’s directions. Reverse transcription of SARS-CoV-2 RNA and Real-Time PCR assay were performed in one-step process. From March to June 2020 analysed targets of SARS-CoV-2 were RdRp, E and N genes (GeneFinder COVID-19 Plus RealAmp Kit, OSANG Healthcare); from July 2020 analysed targets were RdRp and ORF-8 genes (SARS-CoV-2 ELITe MGB Kit).

### Hematological and biochemical investigation

Complete blood count was performed on Sysmex XN-3100 Automatic Hematology Analyzer (Sysmex, Kobe, Japan) based on the fluorescence flow cytometry and hydrodynamically focused impedance.

Serum levels of biochemicals parameters analysed (C-reactive protein, CRP, ferritin, lactate dehydrogenase, LDH, Interleukine-6, IL-6) were performed on the Cobas^®^ 8000 analyser according to manufacturer’s directions (Roche Diagnostic GmbH, Germany).

Plasmatic levels of PCT (procalcitonin) were determined by Enzyme-Linked Fluorescence Assay (ELFA) with VIDAS^®^ B.R.A.H.M.S. PCT™ system (Biomerieux, Marcy-l’Etoile, France) according to manufacturer recommendations and expressed in ng/mL.

D-dimer was quantified by particle-enhanced immunoturbidimetric assay for the quantitative determination of cross-linked fibrin degradation products according to manufacturer recommendations and expressed in ng/mL FEU (Siemens Healthcare GmbH, Erlangen, Germany).

### sFlt-1 and CA 15.3 assay in serum

Serum levels of sFlt-1 and/or CA15.3 were both determined by Electro-Chemi-Luminescence Immuno Assay (ECLIA) fully automated on the immunoanalyzer Elecsys 2010^®^, Cobas 8000, according to manufacturer recommendations (Roche Diagnostic GmbH, Germany). sFlt-1 was expressed in pg/mL and CA 15.3 was expressed in U/mL; all analysed sera were collected and stored at − 80 °C until assay.

### Evaluation of thoracic CT images

Computed Tomography (CT) imaging is the diagnostic tool with the highest sensitivity for COVID-19. In our study, chest CT images of patients were inspected and classified according to a scoring system based on the assessment of grade of pulmonary fibrosis, ground-glass opacities and consolidation extension, and pleural effusion. Lung fibrosis has been categorized as absent (A) or moderate (M), severe (S) or critic (SS) grade, based on the type of lesions and extent of the affected area. The types of lesions were ground-glass opacities, linear opacities, interlobular septal thickening, reticulation, honeycombing and traction bronchiectasis. The extent of each type of lesion was scored based on the number of lung segments affected.

### Statistics

Results are reported as means with standard deviation and interquartiles ranges. The Student’s *t* test was used for comparison between patients and controls; statistically significant differences were established according to *p* value (< 0.05). Receiver Operating Curve (ROC) and the Area Under the Curve (AUC) were used to establish cut-off and decision rules for serum markers associated to COVID-19. Statistical analysis was performed by MedCalc v19.9.1 statistical software.

### Ethics approval and informed consent

All procedures undertaken in this study were in accordance with the ethical standards of the institutional and national research committee as well as the Declaration of Helsinki, its later amendments and other comparable ethical standards. Our study was performed during COVID-19 pandemic on patients and material was collected for diagnostic purposes during patients hospitalisation (secondary use). All leftover serum specimens were anonymised and stored at  − 80 °C until further use; patient’s names and IDs were deleted, and researchers analysed only renumbered anonymous data after association to clinical characteristics. In accordance with the Institutional Review Board of the Vito Fazzi Hospital, where the approved study has been conducted, during COVID-19 pandemic the need for informed consent from the patients was waived due to the urgent need for research insight in the context of this rapidly evolving disease.

### Ethical compliance with human/animal study

All procedures described in the study have been actuated according to ethical principles for medical research involving human subject stated in the Declaration of Helsinki. 

## Results

We investigated and analysed the data regarding serum level of sFlt-1, previously described as a serum marker of endothelial dysfunction during bacterial sepsis and CA 15.3, analog of KL-6, for pulmonary fibrosis, in 262 patients affected by SARS-CoV-2 infection (Table 1).

**Figure 1:**
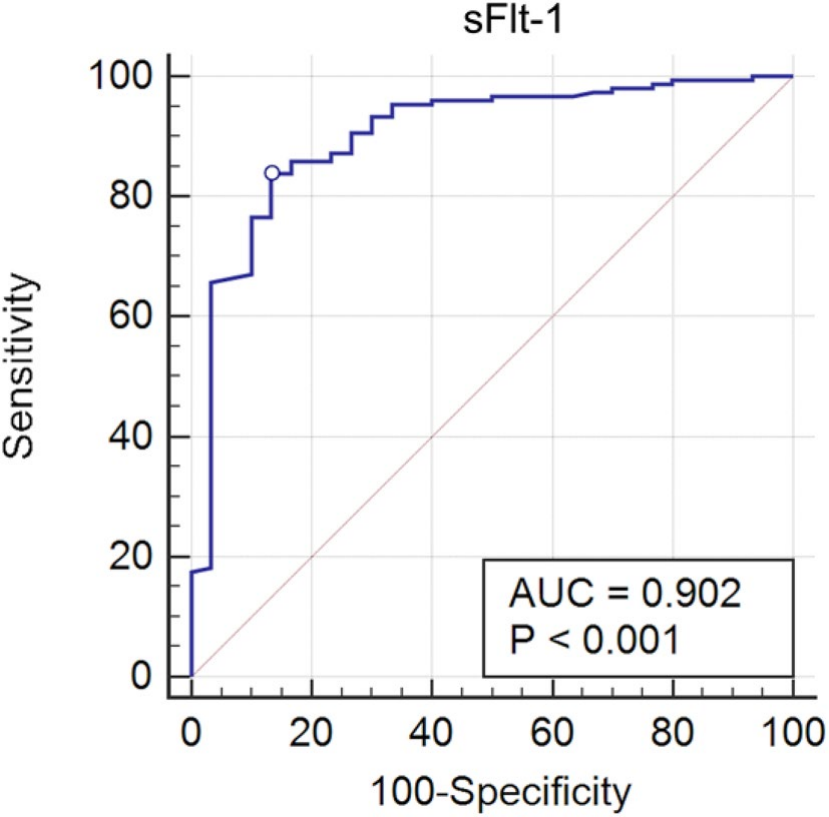
Receiver operating characteristic (ROC) analysis of sFlt-1 serum levels between SARS-CoV-2 infected and non-infected patients; the circle indicates the cut-off point (90.3 pg/mL) with 83.89% Sensitivity and 86.67% Specificity for positive patients. AUC denotes area under the curve.

Serum sFlt-1 showed an association with SARS-CoV-2 infection status with a decisive threshold value for positive patients > 90.3 pg/mL, as resulting from the receiver operating characteristic (ROC) curve (AUC 0.902 with *p* < 0.001, Sensitivity 83.9%, Specificity 86.7%; Fig. 1).

**Figure 2:**
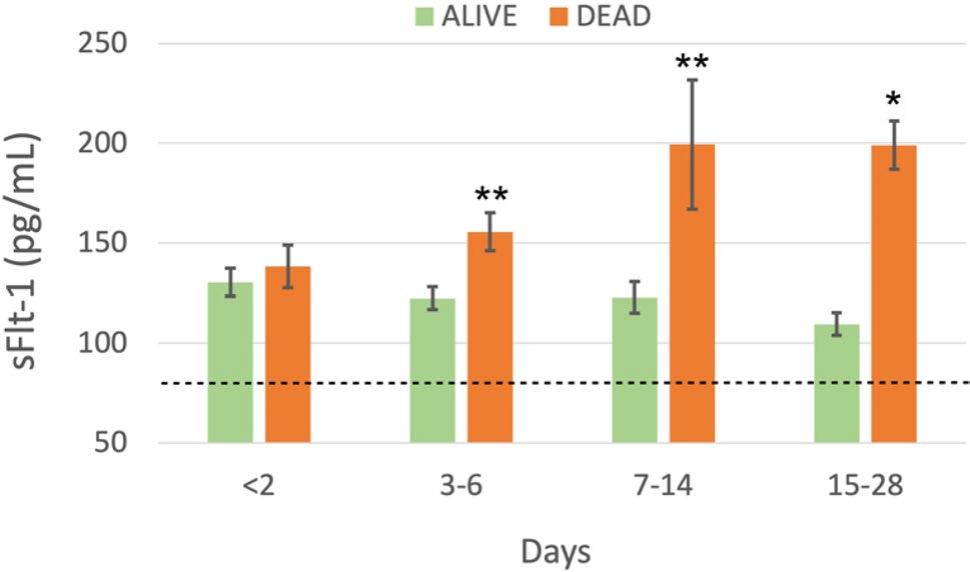
Longitudinal analysis of serum levels of sFlt-1 during hospitalisation of SARS-CoV-2 patients, divided in Alive and Dead according to the outcome of the disease. Patients were monitored for four weeks (less than 2 days, 3–6 days, 7–14 days, 15–28 days from first positive detection of SARS-CoV-2 RNA). The dotted line indicates sFlt-1 level observed in Controls (78.9 ± 2.5 pg/mL); asterisks indicate statistically significant difference: *p < 0.05; **p < 0.01.

Stratifying patients according to the time that passed from the beginning of the disease (first positive swab) and disease outcome, sFlt-1appeared associated with the extent and severity of the disease. A significant increase has been found starting from the third day of hospitalisation in patients with the poorest outcomes (122.4 ± 6.9 vs 155.6 ± 10.6 pg/mL survived vs died, *p* = 0.01), especially, after second week of the disease (up to an average value of 199.4 ± 32.4 pg/mL, at second week, and 199.0 ± 12.0 pg/mL, at third week) in patients with poorest outcome (Fig. 2). In control patients we observed a concentration of 78.9 ± 2.5 pg/mL of sFlt-1.

IL-6, circulating marker associated with extent and severity of the disease, showed a significant increase from the third day of disease in patients with poorest outcome (171.1 ± 68.7 vs 979.4 ± 440.8 pg/mL survived vs died, *p* = 0.005), especially at the second week of disease, and afterwards, (300.5 ± 103.6 vs 1514.2 ± 464.2 pg/mL, *p* < 0.0001).

Values of IL-6 showed an increasing trend in higher sFlt-1 quartiles (Table 2). Moreover, laboratory parameters assayed in COVID-19 patients (CRP, ferritin, LDH, D-dimer, lymphocytes and neutrophils count, RDW-SD, PCT) showed a trend of association with the sFlt-1 interquartile values (Table 2). As reported in Table 2, for poor outcome patients, severe lymphopenia, neutrophilia, increase of RDW-SD were observed for second quartile values of sFlt-1 (99.8–117 pg/mL). RDW-SD (fL) evaluation was used for RDW assessment since it is an independent statistical index, not affected by level of MCV, as the coefficient of variation of red blood cell distribution (RDW-CV)^8^. Severe increase of serum level of CRP, LDH, ferritin and D-dimer were measured in association to third quartile values of sFlt-1 (117.1–142 pg/mL). Patients who survived the virus showed analogous increase of several biochemical parameters (CRP, LDH, ferritin) for fourth quartile values of sFlt-1 (> 142.1 pg/mL).

**Figure 3:**
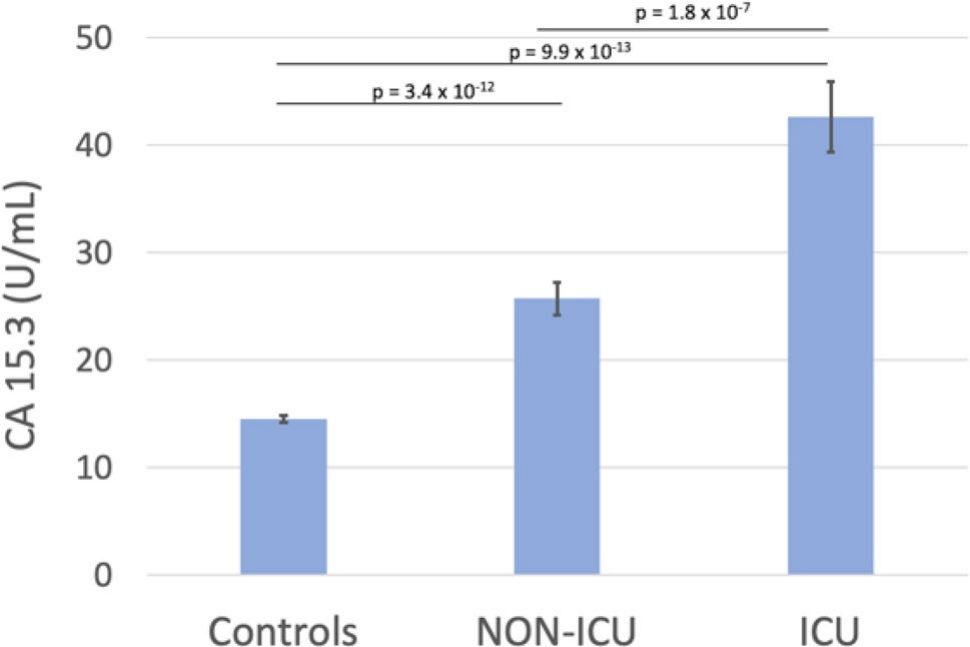
CA 15.3 serum levels in SARS-CoV-2 infected patients, divided according to their hospitalisation in Intensive Care Unit (ICU) or other in different wards (NON-ICU), and control patients.

**Figure 4:**
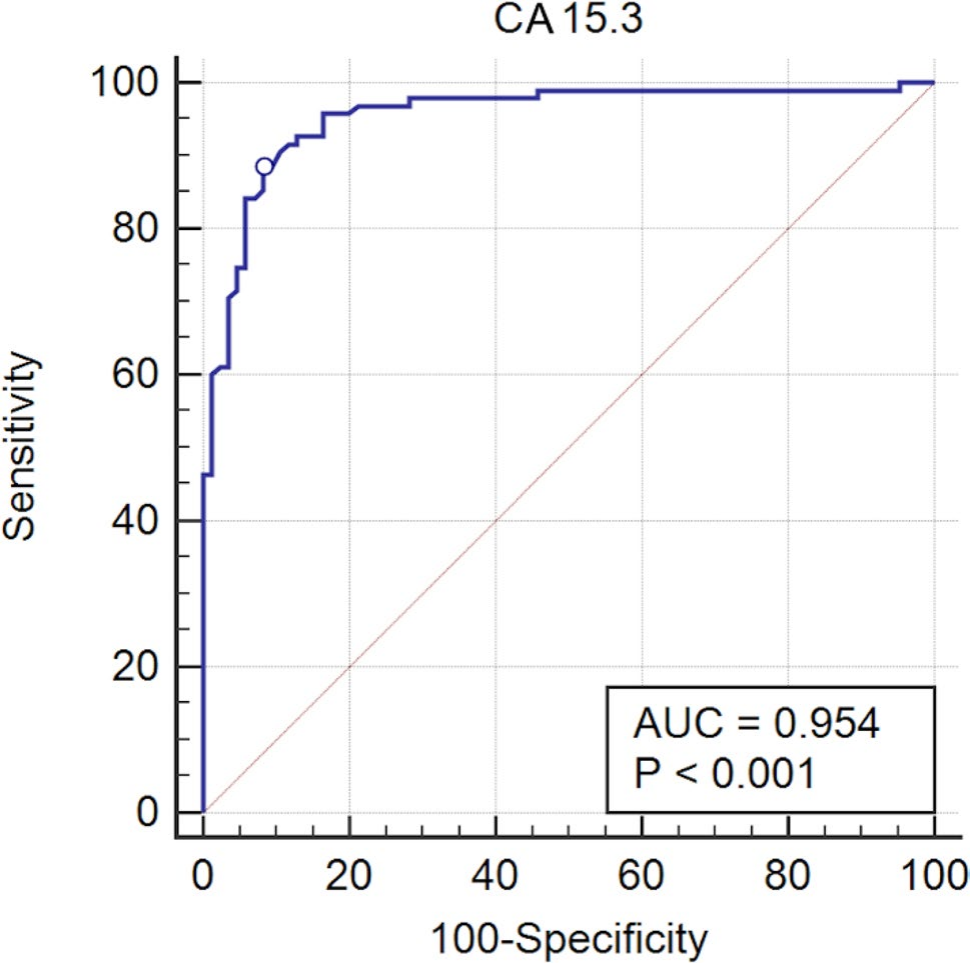
Receiver operating characteristic (ROC) analysis of sFlt-1 serum levels between severe and non-severe SARS-CoV-2 infected patients; the circle indicates the cut-off point (24.8 U/mL) with 88.4% Sensitivity and 91.8% Specificity for positive patients. AUC denotes area under the curve.

We further investigated the possible use of CA 15.3 as marker of pulmonary interstitial damage and progression to fibrosis of the lung in SARS-CoV-2 infection; we found a significant increase of CA 15.3 in SARS-CoV-2 infected patients and a correlation with the severity of clinical conditions. CA 15.3 serum levels of patients hospitalised in Intensive Care ward presented a significant 40% increase compared to patients who were hospitalised in different ward (42.6 ± 3.3 vs 25.7 ± 1.5U/mL, *p* < 0.0001; control patients 14.5 ± 0.3U/mL; Fig. 3)*.* We analysed thoracic CT imaging of the same patients and they presented a severe grade of lung ground-glass opacities, signs of interstitial damage and presence of fibrosis. Although distinguishing between UIP (Usual Interstitial Pneumonia), NSIP (Non-specific Interstitial Pneumonia) and chronic fibrotic hypersensitivity pneumonitis is often difficult due to significant overlap of the radiological features and often requires bronchoalveolar lavage (BAL) examination and/or surgical lung biopsy^20^, in our patients the integrated clinical features and High-Resolution Computed Tomography (HRCT) lesions were in accordance with a prevalent NSIP pattern. Some HRTC findings in our patients may be present in Acute Fibrinous and Organising Pneumonia (AFOP), a histologic entity of lung injury with the hallmark features of intra-alveolar fibrin deposits associated with pneumonia, type II pneumocyte hyperplasia and a patchy lymphohistiocytic proliferations^21^. Although our patients were not submitted to open lung biopsy for the histological diagnosis of their diffuse parenchymal lung disorder, AFOP has been confidently excluded based on the thorough clinical history and extended laboratory diagnostic workup, the shorter duration of the disease, the absence of underlying hematologic disorders and other possible etiologic and risk factors to whom AFOP has been associated to date and the rarity of this entity (Gomes et al. reported just 13 cases of AFOP in a tertiary referral hospital over a period of 14 years)^22^. Correlation of the CT severity grade with the serum level of CA 15.3 of all COVID-19 patients revealed a noteworthy association, the ROC curve analysis exhibited 0.958 AUC value (*p* < 0.0001, Sensitivity 88.4%, Specificity 91.8%) and > 24.8 U/mL cut-off value, which significantly differentiated severe/critic pulmonary interstitial damage in SARS-CoV-2 infection (Fig. 4). Conversely, the absence of pulmonary fibrosis in COVID-19 was associated with a CA 15.3 serum level < 13.9 U/mL (*p* < 0.0001, AUC 0.822, Sensitivity 82.4% and Specificity 83.3%).

**Figure 5:**
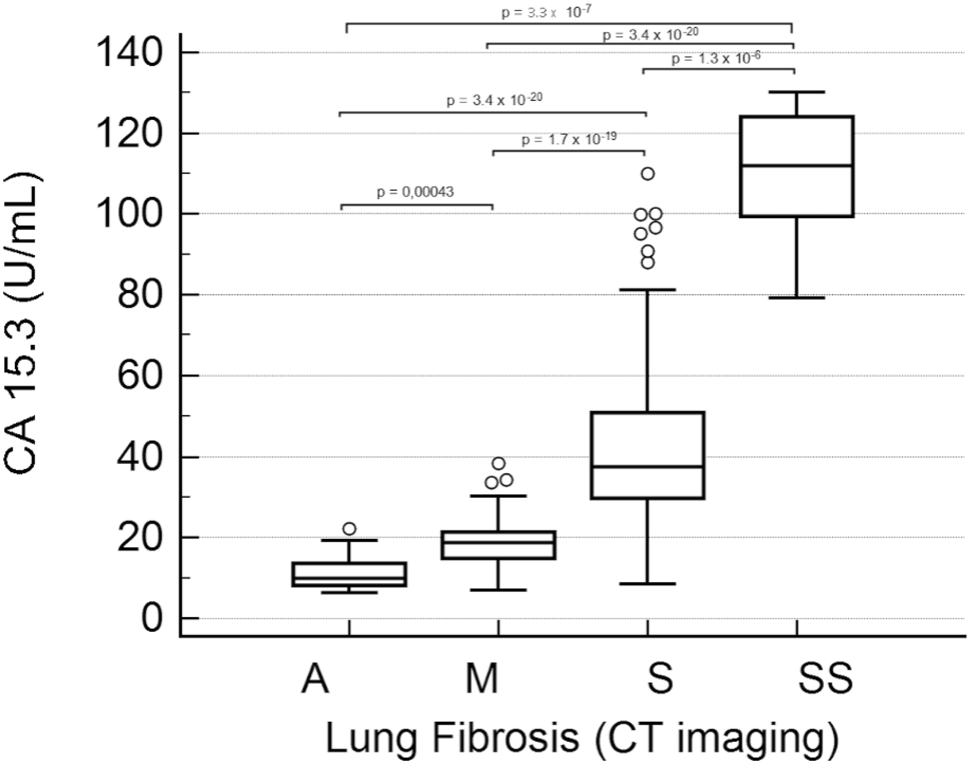
CA 15.3 serum levels in SARS-CoV-2 infected patients according to lung fibrosis and damage severity grade obtained by CT imaging. A, absence of lung fibrosis; M, moderate grade; S, severe grade; SS; critic grade. Boxes represent first and third quartiles (25 to 75 percentile), bands within boxes represent the median value, whiskers display ranges of 1.5 interquartile ranges from the end of the box, outside values are displayed as separate points.

By dividing patients according to the lung fibrosis severity grade CT imaging (i.e., absent, moderate, severe or critic grade of pulmonary ground-glass opacities) a statistically significant increase of serum levels of CA 15.3 was observed in SS and in S patients compared to M patients (i.e., SS, 110.0 ± 6.9 U/mL; S, 43.2 ± 2.2 U/mL; M, 18.4 ± 0.7U/mL and A, 11.6 1.5 U/mL ± 1.5; *p* < 0.0001 in all comparisons; Fig. 5).

## Discussion

We found a significant increase of sFlt-1, a marker of endothelial dysfunction previously associated with bacterial sepsis^12^, in SARS-CoV-2 infected patients studied from March 2020 to February 2021 during COVID-19 pandemic. Decision threshold value for positive patients was > 90.3 pg/mL (Fig. 1), in accordance with previous results (95 pg/mL) found in respiratory failure of COVID-19 patients^13,23^ and with the finding of high sFlt-1 circulating levels in severe disease^14^. In the present study, patients who died during the second or third week of disease, showed significant increase of sFlt-1 serum levels compared to surviving patients at the same week of observation (Fig. 2). In presence of bacterial sepsis previous evidence showed that sFlt-1 values over 200 pg/mL^12^. Libby et al.^24^ defined COVID-19 as an endothelial disease; impaired endothelial barrier function in COVID-19 appears to contribute to protein and fluid accumulation in the alveolar space and impaired oxygenation of the blood. Endothelial dysfunction and consequent uneven balance of prothrombotic/antithrombotic action contribute to thrombosis in situ observed in the pulmonary vasculature of COVID patients^25^ and predispose them to thrombosis events in all arterial beds within the microvasculature, including that of the coronary, kidneys and cerebral circulation. Venous thrombosis and pulmonary embolism commonly complicate COVID-19 and are related to unbalanced endothelial functions^26^. In the present study sFlt-1 higher quartiles were associated with higher concentrations of IL-6 and the worsening of other hematochemical parameters including D-dimer, LDH, Ferritin, CRP, lymphopenia, neutrophilia, RDW-SD, especially when evaluated in severe disease compared to mild or moderate disease (survived patients) (Table2).

RDW reflects the variation of red blood cell (RBC) volume and it implicates an increased rate of RBC destruction, dysfunctional erythropoiesis and/or shortened RBC lifespan which has been suggested like a compensatory up-regulation due to excessive blood hypoxemia^7^ or, as suggested by others, like a consequence of counterregulatory changes of RBC production and turnover due to increased production and turnover of leukocytes or platelets, such as would occur in inflammation^9^. Our results showed a significant increase of RDW-SD in deceased COVID-19 patients compared to those who survived (47.8 fL vs 45.4, *p* = 0.042); highest values for RDW-SD were found in deceased patients with II quartile sFlt-1 levels (Table 2), as observed for leukocytes values (lymphocytes and neutrophils, Table 2). Inflammatory status negatively affects endothelium leading to its dysfunction and, in turn, to impaired angiogenesis and coagulation^25,27^.

In lung tissue from non-survivors with COVID-19, as with influenza, Ackermann et al. showed that microvascular injuries (endothelialitis) and pathologic inflammation induced a significant higher level of intussusceptive angiogenesis with tissue over-expression of pro-angiogenic factors^25^. According to the authors, vascular angiogenesis distinguished the pulmonary pathobiology of COVID-19 from that of equally severe influenza virus infection, moreover the increased degree of intussusceptive angiogenesis appeared significantly correlated with increasing duration of hospitalization. Implication of sFlt-1 in angiogenesis or its competition with membrane bound Flt-1 for binding VEGF and potential role of decoy molecule in the angiogenic process appears an interesting topic for next investigation. In the present study it could not be assessed since patients were not submitted to open lung biopsy for the histological evaluation of angiogenesis.

High serum levels of the inflammatory cytokine IL-6 are associated to the SARS-CoV-2 infection and severity^28–30^. IL-1a and IL-1b, IL-6, and TNF-α, and other components of the inflammatory cascade contribute to host defence against infections although an excessive synthesis determines the cytokines storm, a severe acute systemic inflammatory response which confers increased risks of vascular hyperpermeability, multiorgan failure, and eventually death^31^.

In this context, the potential use of sFlt-1 as a marker of endothelial dysfunction opens the possibility of new diagnostic and treatment strategy of COVID-19 patients.

Lung findings of patients who died with COVID-19 showed that the main pathological feature is an early-phase or intermediate-phase diffuse alveolar damage, marked fibrotic lung parenchymal remodeling, characterized by fibroblast proliferation, airspace obliteration, and micro-honeycombing, in addition to the presence of fibrin thrombi in small arterial vessels^32^. In the present study we correlated the disease severity and lung fibrosis of COVID-19 patients with circulating levels of CA 15.3.

The CA 15.3 has been previously described as alternative marker for KL-6 fibrotic lung disease^33^, a mucin-like glycoprotein mainly expressed on the type II alveolar epithelial cell surface^34^ and as marker of pulmonary fibrosis^35,36^.

Kl-6 is recognized as a prognostic biomarker of Interstitial Lung Disease (ILDs), predicting response to antifibrotic therapies^33,37,38^, has been correlated with risk of mortality in Acute Respiratory Distress Syndrome (ARDS)^39^ and reported as indicator of alveolar epithelial cell damage induced by mechanical ventilatory support^39^. High peripheral levels of this protein have also been reported in Legionella, Pneumocystis jivorecii infections^40,41^ and measles‐associated pneumonia^42^, as well as in viral pneumonia^43^.

Recent evidence indicated serum KL-6 as a biomarker of severe COVID-19, being elevated in severe patients admitted to the Intensive Care Unit (ICU) requiring intubation and mechanical ventilation for diffuse interstitial pneumonia^44^.

Similarly, Deng et al.^45^ described KL-6 as sensitive and specific biomarker for distinguishing mild and severe/critical patients correlated to computed tomography lung lesions areas and predicting the prognosis of lung injury of discharged patients.

Kruit et al.^16^ reported that KL-6 is identical to the target molecule to which antibodies collectively known as CA 15.3 have been developed, obtaining equivalent results for the assay of the two molecules in patients with fibrotic lung disease.

In the present study we observed a very significant association of CA 15.3 with the presence of lung ground-glass opacities and pulmonary lesions typical of thoracic CT imaging of COVID-19 patients with a cut-off value > 24.8 U/mL able to discriminate COVID-19 patients with severe lung fibrosis (Fig. 4).

We found serum levels of CA 15.3 slightly, although significantly, increased in patients with moderate grade of lung fibrosis compared to controls (18.4 ± 0.7 U/mL vs 14.5 ± 2.5 U/mL in M patients vs Controls, *p* < 0.001); no increase was found in COVID-19 patients with no lung fibrosis. On the other hand, severe and critic lung fibrosis were characterized with a marked and significant increase of CA 15.3 (up to 43.2 ± 2.2 and 110.0 ± 6.9 U/mL average value respectively, Fig. 5). ICU hospitalised patients analysed in the present study had circulating levels of CA 15.3 significantly higher than NON-ICU hospitalised patients (42.6 ± 3.3 vs 25.7 ± 1.5 U/mL, *p* < 0.0001, Fig. 3). Although CA 15.3 circulating levels tend to increase during hospitalisation, there is no significant difference between poor and good outcome patients at the same week of observation in the time course of the disease. The opposite behaviour of analysed markers of inflammatory and endothelial damage, IL-6 and sFlt-1, further evidences the significant role of inflammation and endothelial damage in determining the severity of COVID-19.

In conclusion, serum level of sFlt-1 and Ca 15.3 can help categorise clinical phase of COVID-19 patients. From our result, sFlt-1 might identify hospitalised patients with a severe clinical condition related to endothelial dysfunction and consequent clinical sequalae, which could help clinicians to better personalise treatment; CA 15.3 appears a promising biomarker for severity of pulmonary involvement in COVID-19. Further studies on expanded number of patients and extended clinical evaluation would help to better understand clinical utility of these biomarkers.

## Data Availability

The datasets used and/or analyzed during the current study are available from the corresponding author on reasonable request.
